# Human periodontal ligament cells exhibit no endotoxin tolerance upon stimulation with *Porphyromonas gingivalis* lipopolysaccharide

**DOI:** 10.1111/jre.12549

**Published:** 2018-03-26

**Authors:** A. Blufstein, C. Behm, P. Q. Nguyen, X. Rausch‐Fan, O. Andrukhov

**Affiliations:** ^1^ School of Dentistry Medical University of Vienna Vienna Austria

**Keywords:** endotoxin tolerance, lipopolysaccharide, periodontal ligament cells, periodontitis, *Porphyromonas gingivalis*

## Abstract

**Background/Objectives:**

Endotoxin tolerance is characterized by a state of hyporesponsiveness after confrontation with endotoxins such as lipopolysaccharides (LPS) at low concentrations. The aim of this study was to investigate, whether pretreatment with *Porphyromonas gingivalis* leads to endotoxin tolerance induction and possible alterations in toll‐like receptor (TLR) 2‐ and 4‐induced response in human periodontal ligament cells (hPDLCs).

**Material and Methods:**

Primary hPDLCs were pretreated with *P. gingivalis* (0.1 or 0.3 μg/mL) LPS for 24 hours and afterwards treated with one of the following stimuli: *P. gingivalis* LPS (1 μg/mL); TLR4 agonist *Escherichia coli* LPS (0.1 μg/mL; 1 μg/mL); TLR2 agonist Pam3CSK4 (0.1 μg/mL; 1 μg/mL). The protein expression of interleukin (IL)‐6, IL‐8 and monocyte chemotactic protein‐1 was analyzed with quantitative polymerase chain reaction and enzyme‐linked immunosorbent assay. Gene expression levels of TLR2 and TLR4 were determined by quantitative polymerase chain reaction.

**Results:**

Pretreatment of cells with low concentrations of *P. gingivalis* LPS did not result in lower production of IL‐6, IL‐8 and monocyte chemotactic protein‐1 compared to control group. In some cases, pretreated cells exhibited lower gene expression levels of TLR2 and TLR4 compared to non‐pretreated cells.

**Conclusion:**

The results of this study implicate that hPDLCs do not develop endotoxin tolerance. Furthermore, the amplitude of the inflammatory response shows no significant dependency on TLR2 and TLR4 expression levels.

## 1 INTRODUCTION

Endotoxin tolerance is a phenomenon observed in immune cells and other cytokine producing cells to prevent excessive and prolonged inflammatory response.^1^, ^2^ Exposed to low concentrations of endotoxins such as lipopolysaccharide (LPS), these cells shift into a state of hyporesponsiveness. Consequently, a subsequent confrontation with LPS in higher doses results in reduced cytokine production.^3^ In this matter, the endotoxin tolerance provides protection against tissue destruction in multiple pathologies.^4^, ^5^

Periodontitis is a multifactorial, chronic disease leading to the damage of periodontal soft and hard tissue structure and is one of the primary reasons for adult tooth loss.^6^, ^7^ Periodontitis is caused on the one hand by periodontopathogenic bacteria, on the other hand by a dysregulation of the hosts’ inflammatory response to bacterial virulence factors.^8^, ^9^

One of the most important periodontopathogenic agents is LPS of the strict anaerobic, gram‐negative bacterium *Porphyromonas gingivalis*.^10^, ^11^ *P. gingivalis* LPS induces host cell activation via activation of either toll‐like receptor (TLR) 2 and/or TLR4.^12^, ^13^ Some studies suggest that TLR2 activation by *P. gingivalis* LPS might be related in some cases to the impurity of LPS preparations.^14^ TLR4 binding occurs by a CD14‐dependent mechanism, which can be found either as membrane bound or as a soluble form in the serum.^15^ After recognition of LPS, TLRs initiate several signaling pathways resulting in expression of proinflammatory cytokines and activation of hosts’ immune response.^16^

The main cell population responsible for the inflammatory reaction in periodontal disease are leukocytes such as macrophages, neutrophils and lymphocytes.^8^ Even though these cells are known to develop endotoxin tolerance, the inflammatory signs of periodontitis still persist as long as biofilm is present. Considering this fact, Ara et al investigated the inflammatory response of LPS‐pretreated human gingival fibroblasts. Their study demonstrated that human gingival fibroblasts do not induce endotoxin tolerance and thus may have a crucial influence on the progression of periodontal disease.^17^

Human periodontal ligament cells (hPDLCs) are an important component of the periodontal attachment apparatus and have a morphology similar to those of fibroblasts.^18^ These cells are thought to play an important role in the homeostasis of periodontal structures and they share some properties of osteoblasts and cementoblasts.^18^ In addition, these cells are able to produce different inflammatory mediators upon stimulation with bacterial stimuli.^19^ So far there is only one study concerning endotoxin tolerance in hPDLCs by Wu et al suggesting hyporesponsiveness in these cells after previous stimulation.^20^ This finding is rather surprising, because gingival fibroblasts and PDLCs share many common properties.^21^ In addition, in this study stimulation with LPS was performed in the absence of soluble CD14 (sCD14). It is known that hPDLCs lack of membrane bound CD14 protein and sCD14 significantly enhances the response of these cells to bacterial LPS.^22^ Moreover, sCD14 is present in saliva and gingival crevicular fluid and therefore its application in experiments with LPS reflects physiological conditions more adequately.^23^

Therefore, the primary aim of this study was to investigate, whether hPDLCs induce endotoxin tolerance when treated with *P. gingivalis* LPS in low concentrations combined with physiologically relevant levels of sCD14. As *P. gingivalis* LPS activates both TLR2 and TLR4, the secondary aim was to evaluate the receptors separately by treatment with TLR4 agonist *Escherichia coli* LPS and synthetic TLR2 agonist Pam3CSK4.

## 2 MATERIAL AND METHODS

### 2.1 Cell culture

Primary hPDLCs were isolated via the outgrowth method from third molars of six healthy donors, which were extracted due to orthodontic indications. Each patient gave written informed consent before the experiment. PDL tissue attached to the middle third of the root surface was scraped off with a scalpel and reduced to small pieces. The tissue fragments were cultured in petri dishes with Dulbecco's modified Eagle medium (DMEM) supplemented with 10% fetal bovine serum, streptomycin (50 μg/mL) and penicillin (100 U/mL) (Gibco^**®**^, Life Technologies, Carlsbad, CA, USA) and incubated in a humid environment of 5% CO_2_ at 37°C. After the outgrowing cells reached confluence, detachment was performed with accutase. The hPDLCs were seeded in cultural flasks containing DMEM supplemented with fetal bovine serum, streptomycin and penicillin as described above. Cells between passages 3 and 6 were used in the subsequent experiments. Protocol for primary hPDLCs isolation was approved by the Ethics Committee of the Medical University of Vienna.

### 2.2 Study protocol

HPDLCs were seeded in 24‐well plates at a density of 5 × 10^4^ cells per well and incubated for 24 hours. Subsequently, some hPDLCs were pretreated with *P. gingivalis* LPS (0.1 or 0.3 μg/mL) in the presence of soluble CD14 (250 ng/mL) in serum‐free DMEM supplemented with 1% penicillin/streptomycin. As shown by our recent study and preliminary data, stimulation with such *P. gingivalis* LPS concentrations result in a submaximal response in hPDLCs.^22^ Another group of hPDLCs did not get any treatment and were incubated with serum‐free DMEM with 1% penicillin/streptomycin alone. After 24 hours, both pretreated and non‐pretreated cells were washed twice with phosphate‐buffered saline and stimulated with one of the following stimuli: *P. gingivalis* LPS (1 μg/mL), ultrapure *E. coli* LPS (0.1 μg/mL), TLR2 agonist Pam3CSK4 (0.1 and 1 μg/mL) for another 24 hours. *P. gingivalis* LPS and *E. coli* LPS were applied in combination with soluble CD14 (250 ng/mL). Cells incubated with serum‐free DMEM were used as controls. All stimulation reagents were purchased from InvivoGen (San Diego, CA, USA). After stimulation, the expression of different genes and the levels of different proteins in conditioned media were measured by quantitative polymerase chain reaction (qPCR) and enzyme‐linked immunosorbent assay (ELISA), respectively.

### 2.3 Quantitative polymerase chain reaction

Isolation of mRNA and transcription into cDNA was performed using the TaqMan Gene Expression Cells‐to‐CT kit (Ambion/Applied Biosystems, Foster City, CA, USA) as described in our previous studies.^24^, ^25^, ^26^ The mRNA expression levels of interleukin (IL)‐6, IL‐8, monocyte chemotactic protein (MCP)‐1, TLR2 and TLR4 were determined by qPCR with the GAPDH encoding gene as internal reference. An ABI Prism SDS 7000 device (Applied Biosystems) was used for qPCR with Taqman^®^ gene expression assays with the following ID numbers (all from Applied Biosystems): IL‐6, Hs00985639_m1; IL‐8, Hs00174103_m1; MCP‐1 Hs00234140_m1; TLR2, Hs00610101_m1; TLR4, Hs00152939_m1.

The reactions were carried out in duplicates with the following thermocycler settings: 95°C for 10 minutes, 40 cycles, each for 15 seconds at 95°C and for 1 minute at 60°C. For each sample, the point at which the PCR product reached a defined threshold (cycle threshold *C*_t_) was determined. ΔΔ*C*_t_ values were calculated according to following formula: ΔΔ*C*_t_ = (*C*_t_^target^ – *C*_t_^GAPDH^)_sample_ – (*C*_t_^target^ – *C*_t_^GAPDH^)_control_. The difference in the expression of target genes was calculated with the 2^−ΔΔ*C*t^ method with a control sample. Cells that were not pretreated with *P. gingivalis* LPS and were not stimulated with any stimuli were used as control.

### 2.4 Enyzme‐linked immunosorbent assay

After the second stimulation and incubation, the supernatants were collected to determine the protein concentration of IL‐6, IL‐8 and MCP‐1 with ELISA using commercially available ELISA Ready‐Set‐Go! kits (eBioscience, San Diego, CA, USA) according to the manufacturer's instructions. The assay ranges of different kits are stated as follows: IL‐6, 2‐200 pg/mL; IL‐8, 2‐250 pg/mL; MCP‐1, 7‐1000 pg/mL. Therefore, samples stimulated with Pam3CSK4 were diluted 20‐fold, *P. gingivalis* LPS groups were diluted 4‐fold, control groups and samples with *E. coli* LPS treatment remained undiluted. The samples were applied in duplicates. The optical densities were plotted against a standard curve and concentrations were stated as picograms per milliliter.

### 2.5 Statistical analysis

The statistical analysis was executed with the statistical program spss 21.0 (IBM Corp., Armonk, NY, USA). A Kolmogorov‐Smirnov test was performed to test the normal distribution of all data. Statistical differences between different groups were analyzed by one‐way analysis of variance for repeated measures followed by *t*‐test. Data are expressed as mean ± SEM. Differences were considered to be statistically significant at *P *<* *.05.

## 3 RESULTS

### 3.1 Effect of human periodontal ligament cell pretreatment with 0.1 μg/mL *P. gingivalis* lipopolysaccharide on their response to different stimuli

**Figure 1:**
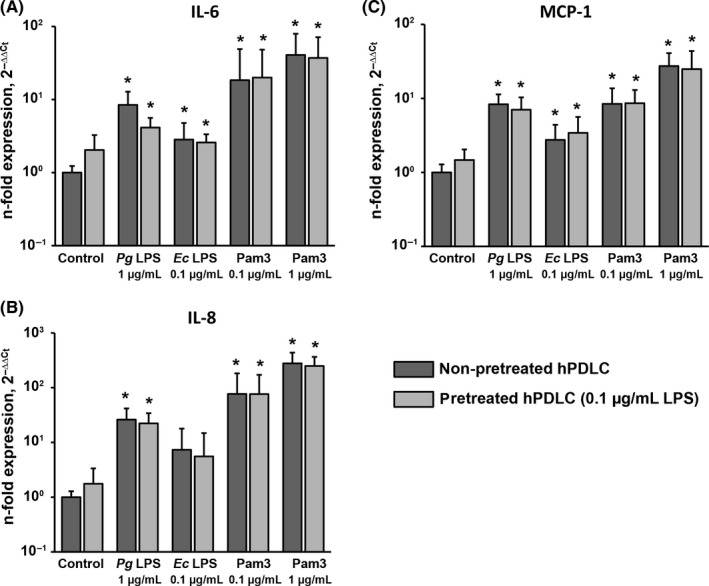
Effect of hPDLC pretreatment with 0.1 μg/mL *Pg* LPS on gene expression levels of IL‐6, IL‐8 and MCP‐1 in response to different stimuli. Gene expression levels of IL‐6 (A), IL‐8 (B) and MCP‐1 (C) were measured by quantitative polymerase chain reaction upon stimulation with *Pg* LPS,* Ec* LPS and Pam3CSK4 in hPDLCs pretreated with 0.1 μg/mL *Pg* as well as in non‐pretreated cells. Both LPS were used in combination with recombinant sCD14 (250 ng/mL). Y‐axes represent the n‐fold expression levels of target gene in relation to non‐pretreated non‐stimulated cells. Data are presented as mean ± SEM of 6 independent experiments on 6 different donors. *Significantly higher vs control (non‐pretreated cells), *P *<* *.05. *Ec*,* Escherichia coli*; hPDLC, human periodontal ligament cells; IL, interleukin; LPS, lipopolysaccharide; MCP, monocyte chemotactic protein; *Pg*,* Porphyromonas gingivalis*

Figure 1 shows the effect of hPDLC pretreatment with 0.1 μg/mL *P. gingivalis* LPS on the gene expression levels of IL‐6, IL‐8 and MCP‐1 induced by their subsequent stimulation with *P. gingivalis* LPS, *E. coli* LPS and Pam3CSK4. No significant difference in the gene expression levels of IL‐6, IL‐8 and MCP‐1 between pretreated and non‐pretreated cells was observed in the control group. Stimulation with all stimuli resulted in a significant increase in the expression levels of all proteins compared to the control group. No significant difference in IL‐6, IL‐8 and MCP‐1 gene expression was observed between pretreated and non‐pretreated hPDLCs in their response to *P. gingivalis* LPS, *E. coli* LPS and Pam3CSK4.

**Figure 2:**
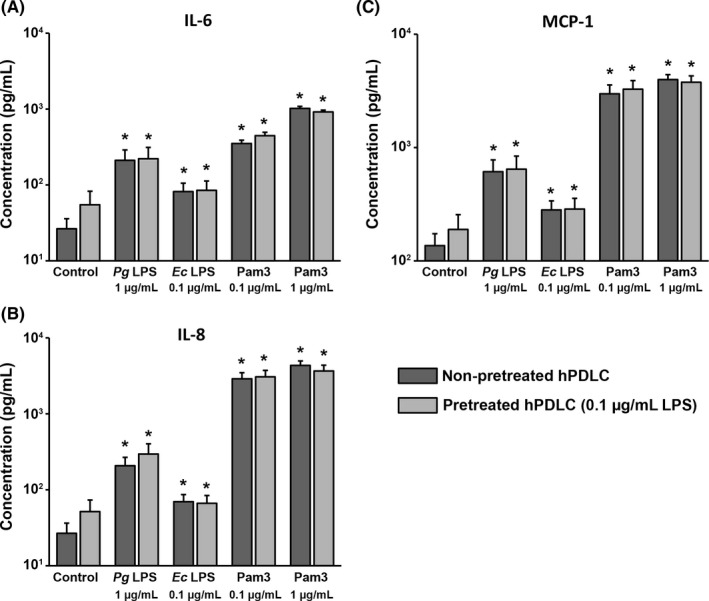
Effect of hPDLC pretreatment with 0.1 μg/mL *Pg* LPS on concentration of IL‐6, IL‐8 and MCP‐1 in conditioned media of hPDLCs after stimulation with different stimuli. Concentration of IL‐6 (A), IL‐8 (B) and MCP‐1 (C) were measured by enzyme‐linked immunosorbent assay in conditioned media of hPDLCs upon stimulation with *Pg* LPS,* Ec* LPS and Pam3CSK4 in hPDLCs pretreated with 0.1 μg/mL *Pg* as well as in non‐pretreated cells. Both LPS were used in combination with recombinant sCD14 (250 ng/mL). Data are presented as mean ± SEM of 6 independent experiments on 6 different donors. *Significantly higher vs control (non‐pretreated cells), *P *<* *.05. *Ec*,* Escherichia coli*; hPDLC, human periodontal ligament cells; IL, interleukin; LPS, lipopolysaccharide; MCP, monocyte chemotactic protein; *Pg*,* Porphyromonas gingivalis*

Figure 2 shows the effect of hPDLC pretreatment with 0.1 μg/mL *P. gingivalis* LPS on the concentration of IL‐6, IL‐8 and MCP‐1 proteins in conditioned media induced by their subsequent stimulation with different stimuli. In the control group, the level of all mediators was higher in conditioned media of pretreated cells compared to those of non‐pretreated cells, but these differences were not statistically significant. All stimuli induced a significant increase in the production of IL‐6, IL‐8 and MCP‐1 proteins by hPDLCs. No significant difference in IL‐6, IL‐8 and MCP‐1 protein production was observed between pretreated and non‐pretreated cells upon stimulation with any stimuli.

**Figure 3:**
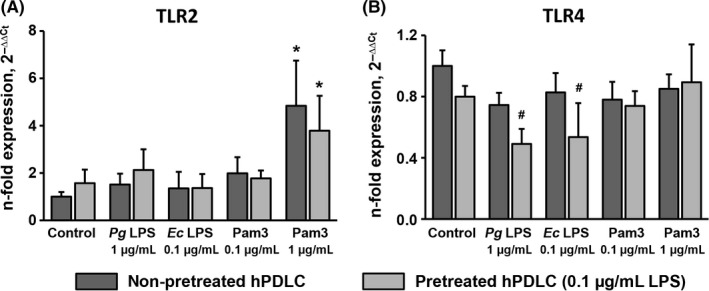
Effect of hPDLC pretreatment with 0.1 μg/mL *Pg* LPS on gene expression levels of TLR2 and TLR4 in hPDLCs in response to different stimuli. Gene expression levels of TLR2 (A) and TLR4 (B) were measured by quantitative polymerase chain reaction upon stimulation with *Pg* LPS,* E. coli* LPS and Pam3CSK4 in hPDLCs pretreated with 0.1 μg/mL *Pg* as well as in non‐pretreated cells. Both LPS were used in combination with recombinant sCD14 (250 ng/mL). Y‐axes represent the n‐fold expression levels of target gene in relation to non‐pretreated non‐stimulated cells. Data are presented as mean ± SEM of 6 independent experiments on 6 different donors. *Significantly higher vs control (non‐pretreated cells), *P *<* *.05. #Significantly different between pretreated and non‐pretreated cells, *P *<* *.05. *Ec*,* Escherichia coli*; hPDLC, human periodontal ligament cells; LPS, lipopolysaccharide; *Pg*,* Porphyromonas gingivalis*; TLR, toll‐like receptor

The gene expression levels of TLR2 and TLR4 in hPDLCs after stimulation with *P. gingivalis* LPS, *E. coli* LPS and Pam3CSK4 in cells pretreated with 0.1 μg/mL *P. gingivalis* LPS and non‐pretreated cells are shown in Figure 3. Gene expression levels of TLR2 were significantly increased upon stimulation with Pam3CSK4 in concentration of 1 μg/mL. No significant difference in the TLR2 gene expression levels between pretreated and non‐pretreated cells was observed. In contrast, some differences in TLR4 expression between pretreated and non‐pretreated cells were observed. Particularly, upon stimulation with *P. gingivalis* LPS and *E. coli* LPS, gene expression levels of TLR4 was significantly lower in pretreated cells compared to non‐pretreated cells. No significant difference in TLR4 expression between pretreated and non‐pretreated cells was observed upon stimulation with Pam3CSK4.

### 3.2 Effect of human periodontal ligament cell pretreatment with 0.3 μg/mL *P. gingivalis* lipopolysaccharide on their response to different stimuli

**Figure 4:**
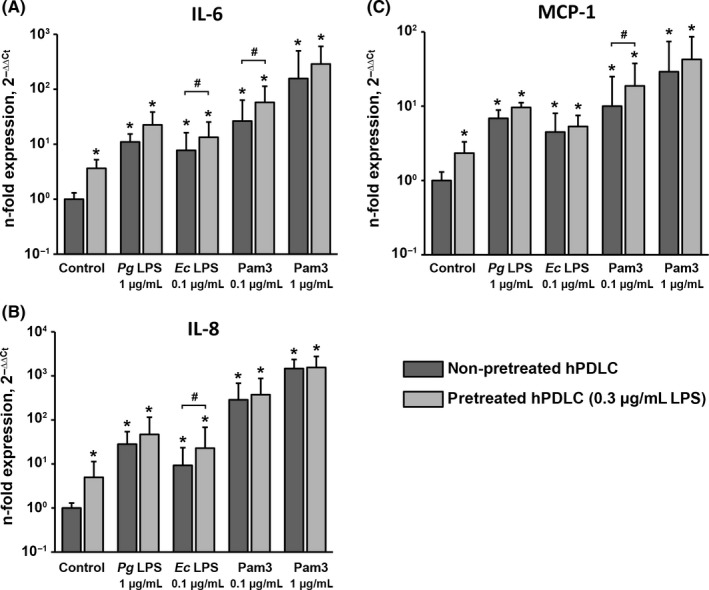
Effect of hPDLC pretreatment with 0.3 μg/mL *Pg* LPS on gene expression levels of IL‐6, IL‐8 and MCP‐1 in response to different stimuli. Gene expression levels of IL‐6 (A), IL‐8 (B) and MCP‐1 (C) were measured by quantitative polymerase chain reaction upon stimulation with *Pg* LPS,* Ec* LPS and Pam3CSK4 in hPDLCs pretreated with 0.3 μg/mL *Pg* as well as in non‐pretreated cells. Both LPS were used in combination with recombinant sCD14 (250 ng/mL). Y‐axes represent the n‐fold expression levels of target gene in relation to non‐pretreated non‐stimulated cells. Data are presented as mean ± SEM of 6 independent experiments on 6 different donors. *Significantly higher vs control (non‐pretreated cells), *P *<* *.05. #Significantly different between pretreated and non‐pretreated cells, *P *<* *.05. *Ec*,* Escherichia coli*; hPDLC, human periodontal ligament cells; IL, interleukin; LPS, lipopolysaccharide; MCP, monocyte chemotactic protein; *Pg*,* Porphyromonas gingivalis*

Figure 4 shows the effects of hPDLC pretreatment with 0.3 μg/mL *P. gingivalis* LPS on the gene expression levels of IL‐6, IL‐8 and MCP‐1 induced by their subsequent stimulation with *P. gingivalis* LPS, *E. coli* LPS and Pam3CSK4. In the control group, pretreatment with 0.3 μg/mL *P. gingivalis* LPS resulted in significantly higher gene expression levels of all proinflammatory mediators. Stimulation with all stimuli resulted in a significant increase in the expression level of all proteins compared to the control group. Stimulation with all stimuli induced generally higher gene expression levels of all proinflammatory mediators in pretreated compared to non‐pretreated cells. Significant differences were observed for IL‐6 upon stimulation with *E. coli* LPS and Pam3CSK4 (0.1 μg/mL), for IL‐8 upon stimulation with *E. coli* LPS and for MCP‐1 upon stimulation with Pam3CSK4 (0.1 μg/mL).

**Figure 5:**
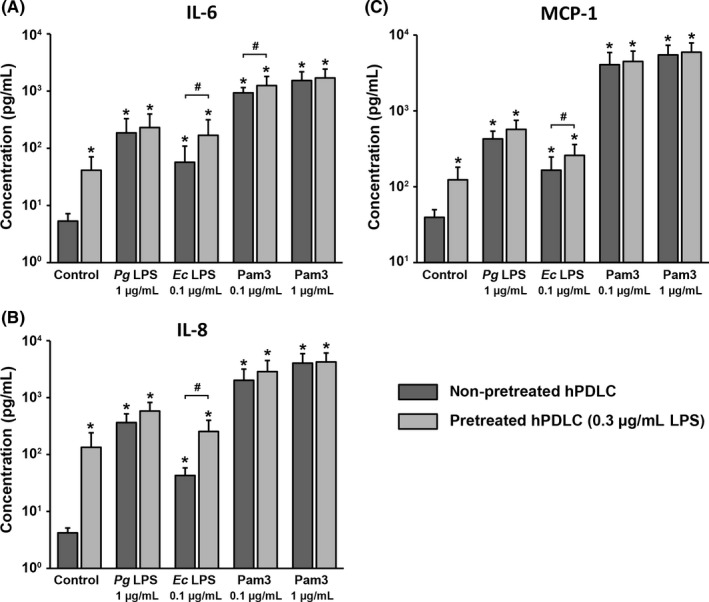
Effect of hPDLC pretreatment with 0.3 μg/mL *Pg* LPS on concentration of IL‐6, IL‐8, and MCP‐1 in conditioned media of hPDLCs after stimulation with different stimuli. Concentration of IL‐6 (A), IL‐8 (B) and MCP‐1 (C) were measured by enzyme‐linked immunosorbent assay in conditioned media of hPDLCs upon stimulation with *Pg* LPS,* Ec* LPS and Pam3CSK4 in hPDLCs pretreated with 0.3 μg/mL *Pg* as well as in non‐pretreated cells. Both LPS were used in combination with recombinant sCD14 (250 ng/mL). Data are presented as mean ± SEM of 6 independent experiments on 6 different donors. *Significantly higher vs control (non‐pretreated cells), *P *<* *.05. #Significantly different between pretreated and non‐pretreated cells, *P *<* *.05. *Ec*,* Escherichia coli*; hPDLC, human periodontal ligament cells; IL, interleukin; LPS, lipopolysaccharide; MCP, monocyte chemotactic protein; *Pg*,* Porphyromonas gingivalis*; TLR, toll‐like receptor

Figure 5 shows the effects of hPDLC pretreatment with 0.3 μg/mL *P. gingivalis* LPS on the concentration of IL‐6, IL‐8 and MCP‐1 induced by their subsequent stimulation with *P. gingivalis* LPS, *E. coli* LPS and Pam3CSK4. In the control group, pretreatment with 0.3 μg/mL *P. gingivalis* LPS resulted in significantly higher protein levels of all proinflammatory mediators. Stimulation with all stimuli resulted in a significant increase in the levels of all proteins compared to the control group. Stimulation with all stimuli generally resulted in higher protein production in pretreated compared to non‐pretreated cells. Significant differences were observed for IL‐6 upon stimulation with *E. coli* LPS and Pam3CSK4 (0.1 μg/mL) as well as for IL‐8 and MCP‐1 upon stimulation with *E. coli* LPS.

**Figure 6:**
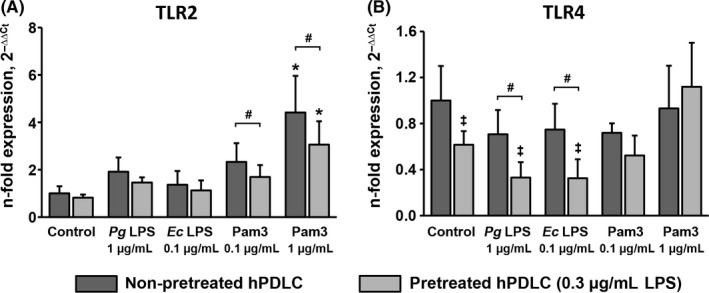
Effect of hPDLC pretreatment with 0.3 μg/mL *Pg* LPS on gene expression levels of TLR2 and TLR4 in hPDLCs in response to different stimuli. Gene expression levels of TLR2 (A) and TLR4 (B) were measured by quantitative polymerase chain reaction upon stimulation with *Pg* LPS,* Ec* LPS and Pam3CSK4 in hPDLCs pretreated with 0.3 μg/mL *Pg* as well as in non‐pretreated cells. Both LPS were used in combination with recombinant sCD14 (250 ng/mL). Y‐axes represent the n‐fold expression levels of target gene in relation to non‐pretreated non‐stimulated cells. Data are presented as mean ± SEM of 6 independent experiments on 6 different donors. *Significantly higher vs control (non‐pretreated cells), *P *<* *.05. ‡Significantly lower vs control (non‐pretreated cells), *P *<* *.05. #Significantly different between pretreated and non‐pretreated cells, *P *<* *.05. *Ec*,* Escherichia coli*; hPDLC, human periodontal ligament cells; LPS, lipopolysaccharide; *Pg*,* Porphyromonas gingivalis*; TLR, toll‐like receptor

The gene expression levels of TLR2 and TLR4 in hPDLCs after stimulation with *P. gingivalis* LPS, *E. coli* LPS and Pam3CSK4 in cells pretreated with 0.3 μg/mL *P. gingivalis* LPS and non‐pretreated cells is shown in Figure 6. Gene expression levels of TLR2 were significantly increased upon stimulation with Pam3CSK4 at a concentration of 1 μg/mL. Cells pretreated with 0.3 μg/mL *P. gingivalis* LPS exhibited significantly lower TLR2 gene expression levels upon stimulation with Pam3CSK4 compared to non‐pretreated cells. Pretreated cells exhibited significantly lower TLR4 gene expression levels in the non‐stimulated group as well as upon stimulation with *P. gingivalis* LPS and *E. coli* LPS compared to non‐pretreated cells. No significant difference in TLR4 expression between pretreated and non‐pretreated cells was observed upon stimulation with Pam3CSK4.

## 4 DISCUSSION

This study addresses the question whether hPDLCs develop endotoxin tolerance after pretreatment with *P. gingivalis* LPS at a concentration inducing submaximal response. To achieve this aim, hPDLCs of six healthy donors were pretreated with *P. gingivalis* LPS at a concentration of 0.1 or 0.3 μg/mL. These concentrations trigger a submaximal inflammatory response in hPDLCs, as determined in preliminary experiments and shown in our previous report.^22^ Pretreatment was performed for 24 hours based on our own experience and published study^27^ showing that the response of hPDLCs to *P. gingivalis* LPS reaches a maximum after 24 hours. After pretreatment, further stimulation with *P. gingivalis* LPS at a concentration of 1 μg/mL was performed. Previous studies suggest that *P. gingivalis* LPS might activate both TLR2 and TLR4 signaling, which is due to either its heterogeneous nature or insufficient purity. Therefore, we have additionally evaluated a possible alteration in the TLR2‐ and TLR4‐dependent response in hPDLCs upon pretreatment with low concentrations of *P. gingivalis* LPS. For this reason, the response to TLR2/1 agonist Pam3CSK4 and TLR4 agonist *E. coli* LPS was compared between pretreated and non‐pretreated cells. Additionally, both *P. gingivalis* LPS and *E. coli* LPS were administered together with sCD14 in a physiologically relevant concentration.^22^

The response of hPDLCs to different stimuli was evaluated based on the production of IL‐6, IL‐8 and MCP‐1. IL‐6 is a proinflammatory cytokine that induces bone resorption and plays a major role in acute inflammation.^28^ The chemoattractants IL‐8 and MCP‐1 are of great importance for immunopathogenesis of periodontal disease.^29^ Furthermore, gene expression levels of TLR2 and TLR4 were measured. TLRs pertain to a family of transmembrane proteins that are involved in recognition of invading pathogens.^30^ The expression of TLR2 and TLR4 is altered in periodontal disease and therefore these receptors are discussed to play an important role in disease progression.^31^ TLR4 activation by different bacterial LPS induces the production of inflammatory mediators in hPDLCs.^22^ Similarly, TLR2 activation results in cytokine production by PDLs^32^ and gingival fibroblasts.^33^

Endotoxin tolerance is classically defined as phenomenon when cells pretreated with submaximal LPS stimuli exhibit a lower response to further stimuli with a higher LPS concentration compared to non‐pretreated cells. This phenomenon is an important ability of the host to protect itself from damage associated with an excessive immune response.^4^ The results of this study did not show any significant decrease of cytokine expression after all applied stimuli in pretreated cells compared to the non‐pretreated group. This was observed for cells pretreated with two different submaximal concentrations of *P. gingivalis* LPS. This finding is also in agreement with the previous study of Wu et al showing that pretreatment of hPDLCs with a lower LPS concentration does not lead to a decrease in their response to higher LPS concentrations.^20^ Therefore, according to these data we can conclude that hPDLCs do not induce endotoxin tolerance. It can thus be suggested, that hPDLCs may play a crucial role in the progression of periodontal disease, as proposed similarly for human gingival fibroblasts.^17^

Although hPDLCs do not develop the classical endotoxin tolerance state, they do possess an ability to protect themselves during prolonged stimulation. In a previous study Wu et al show that pretreatment of hPDLCs with 1 μg/mL for 24 hours leads to a decreased response of these cells to their subsequent stimulation with similar LPS concentrations. Therefore, the possibility that hPDLCs can adapt the amplitude of their response to bacterial LPS and prevent an excessive response cannot be excluded, but it depends on LPS concentrations and stimulation time. However, the ability of hPDLCs to adapt and dampen the amplitude of their response to LPS seems to be rather limited compared to immune cells.

We have observed that pretreatment of hPDLCs with the 0.3 μg/mL *P. gingivalis* LPS induced significant increase in production of all proinflammatory mediators, even if cells were not subjected to any subsequent stimuli. Moreover, pre‐stimulated cells exhibited, in some cases, a higher response to some stimuli. In particular, pretreated cells exhibited a higher response to *E. coli* LPS and submaximal concentration of Pam3CSK4. This observation can be explained by the fact that we used a standard LPS preparation, which activates both TLR2 and TLR4 receptors. We assume that both receptors are partially activated in pretreated cells, which results in a higher response to pure TLR4 agonist *E. coli* LPS and a submaximal concentration of pure TLR2 agonist Pam3CSK4. These data further confirm that pretreatment with LPS does not diminish the response to any subsequent stimuli. Moreover, the response to inflammatory stimuli might be sometimes enhanced by pretreatment, which is further evidence for the important role of hPDLCs in sustained inflammation. It should be mentioned that in some cases the increase of the hPDLC response by *P. gingivalis* LPS pretreatment was rather small and therefore its biological relevance is not obvious.

Another important finding of this study was that pretreatment of hPDLCs with *P. gingivalis* LPS had no significant effect on their response to TLR2 and TLR4 agonists. Both TLR2 and TLR4 are largely involved in progression of periodontal disease. TLR4 activation by different bacterial LPS induces production of inflammatory mediators in hPDLCs.^22^ Similarly, TLR2 activation results in cytokine production by PDLCs^32^ and gingival fibroblasts.^33^ Our finding is a further indication that hPDLCs cannot develop any immune tolerance state and this is additional evidence of their role in sustained inflammation in periodontal disease.

Interestingly, the response of hPDLCs to bacterial LPS was not diminished in pretreated compared to non‐pretreated cells despite the fact that pretreated cells had significantly lower TLR4 expression levels compared to non‐pretreated cells. TLR4 is thought to be the most relevant receptor for LPS.^34^ However, it seems that the expression level of this receptor had no significant impact on the intensity of inflammatory response in hPDLCs. However, although the changes in the TLR4 expression levels observed in our study were significant, their range was rather small. Therefore, additional studies between TLR4 expression levels and the intensity of the hPDLC responses to bacterial LPS are required.

hPDLC pretreatment with the higher *P. gingivalis* LPS concentration resulted in lower TLR2 gene expression levels compared to non‐pretreated cells. However, decreased TLR2 expression levels did not result in a lower production of proinflammatory mediators. TLR2 expression also was significantly increased upon stimulation with TLR2 agonist Pam3CSK4. This observation is in agreement with our previous study on human gingival fibroblasts.^33^ TLR2 seems to play an important role in progression of periodontal disease and particularly in physiology of hPDLCs. Data of the present study show that the response of hPDLCs to TLR2 is significantly higher than that to bacterial LPS, which is in agreement with our previous reports.^22^, ^26^ Therefore, detailed examination of TLR2 induced response and its potential modulation might be important for understanding pathogenesis of periodontal disease and development of new treatment modalities.

In conclusion, our data show that hPDLCs exhibit no endotoxin tolerance and therefore might play an important role in sustained inflammation in periodontal disease. These cells exhibit a rather low ability to dampen their own inflammatory response, which might depend on the amplitude and duration of inflammatory stimuli. The results of this in vitro study can only be transferred into an in vivo situation in a limited way. In vivo hPDLCs are exposed to numerous cytokines and growth factors and continuously interact with other cell types. Another limitation of this study was the small number of donors involved in the study. A possibility to extend this study is to expand the number of donors and include hPDLCs isolated from patients with periodontitis.

## CONFLICT OF INTEREST

The authors declare no conflict of interest. The founding sponsors had no role in the design of the study; in the collection, analyses, or interpretation of data; in the writing of the manuscript, and in the decision to publish the results.
